# Revisiting Neil Armstrongs Moon-Landing Quote: Implications for Speech Perception, Function Word Reduction, and Acoustic Ambiguity

**DOI:** 10.1371/journal.pone.0155975

**Published:** 2016-09-07

**Authors:** Melissa M. Baese-Berk, Laura C. Dilley, Stephanie Schmidt, Tuuli H. Morrill, Mark A. Pitt

**Affiliations:** 1 Department of Linguistics, University of Oregon, Eugene, Oregon, United States of America; 2 Department of Communicative Sciences and Disorders, Michigan State University, East Lansing, Michigan, United States of America; 3 Program in Linguistics, Department of English, George Mason University, Fairfax, Virginia, United States of America; 4 Department of Psychology, The Ohio State University, Columbus, Ohio, United States of America; University of Kent, UNITED KINGDOM

## Abstract

Neil Armstrong insisted that his quote upon landing on the moon was misheard, and that he had said *one small step for a man*, instead of *one small step for man*. What he said is unclear in part because function words like *a* can be reduced and spectrally indistinguishable from the preceding context. Therefore, their presence can be ambiguous, and they may disappear perceptually depending on the rate of surrounding speech. Two experiments are presented examining production and perception of reduced tokens of *for* and *for a* in spontaneous speech. Experiment 1 investigates the distributions of several acoustic features of *for* and *for a*. The results suggest that the distributions of *for* and *for a* overlap substantially, both in terms of temporal and spectral characteristics. Experiment 2 examines perception of these same tokens when the context speaking rate differs. The perceptibility of the function word *a* varies as a function of this context speaking rate. These results demonstrate that substantial ambiguity exists in the original quote from Armstrong, and that this ambiguity may be understood through context speaking rate.

## Introduction

“That’s one small step for man, one giant leap for mankind.[1]” Neil Armstrong insisted for years that his famous quote upon landing on the moon was misheard, and that he had said “one small step for *a* man” [2]. The addition of *a* critically changes the meaning of the overall utterance, since without it, both *man* and *mankind* share the same meaning. In some cases of misunderstood or misreported quotes, examining the source recording can provide evidence for what was actually said. In this case, however, the controversy has continued, as acoustic examinations of the waveforms and spectrograms generated from the sound files of his transmission have yielded mixed opinions about whether he produced the phrase *for a* or the singular word *for* [3]. Unfortunately, conclusive evidence for or against Armstrong’s claim that he spoke the word *a* is unlikely. However, it is possible to examine the feasibility of his claim from a speech production standpoint, and examine why perception may have differed from his intended target. In the present study, we describe results from two experiments designed to investigate these questions.

The disagreement about what Armstrong said during his transmission from the moon stems partly from several acoustic and spectral characteristics of function words like *a*; they can be quite short in casual speech, consist of just a few pitch periods, and be spectrally indistinguishable from the preceding context [4,5]. This phenomenon is known more broadly as *speech reduction*. Very short, spectrally indistinguishable stretches of speech are extremely frequent in casual speech [6], and can result in highly variable perceptions among listeners for the same stretch of speech [7]. In particular, when speakers’ function words are coarticulated with the preceding syllable(s), it may be more difficult for the listener to perceive the intended word sequence. Therefore, it is possible that an intended production of *for* and an intended production of *for a* may have identical, or nearly identical, acoustic signatures. The ambiguity caused by severe reduction could therefore be the cause of the disagreement among the public as to what exactly Armstrong said. If the two productions are indeed acoustically quite similar, how do listeners disambiguate them?

Whether listeners hear an extra word or not (e.g., *for a* versus *for*) represents an example of well-known problems in research on speech perception: word segmentation, and lexical access. Specifically, it is unclear how human listeners know how many word boundaries are in speech material, locations of word boundaries relative to acoustic cues, and how the boundary-delimited chunks of speech correspond to words in the lexicon [8,9]. Reduction, which is extremely frequent in casual speech [6], can result in highly variable perceptions among listeners for the same speech signal (e.g., 7). When speakers’ function words are coarticulated with the preceding syllable(s), it may be more difficult for the listener to perceive the intended target, which could be the cause of the mismatch between Armstrong’s intended production and the public perception of the statement. This mismatch between an intended, reduced token and the perception of the token can be viewed as a case study for a current important problem in psycholinguistics and speech processing more broadly: how reduced speech, which may vary substantially from a “full” form, is perceived by listeners. A growing body of work is addressing this problem in both perception and production [6,10–13]. In the current experiments, we examine the role of timing in perception and production of reduced tokens, using Neil Armstrong’s moon landing quote as the inspiration.

The distal speech rate, or the speech rate of the portion of the utterance surrounding the co-articulated region, influences whether a coarticulated function word is heard [14–19]. If a listener hears slower speech, he or she is likely expect to hear fewer syllables in upcoming material. Therefore, an ambiguous region that could contain one or two syllables is heard as containing one syllable when the surrounding speech rate is relatively slow, and two syllables when the surrounding speech is relatively fast. Therefore, reduced syllables, including function words, are significantly less likely to be heard when distal context is slowed [14]. That is, when the context speech rate is relatively slow compared to the co-articulated region (or conversely if the co-articulated region is spoken relatively fast compared with the context speech rate) it is likely that the listener will not perceive a function word, even if it was produced in the original utterance. In the case of Neil Armstrong, he spoke the phrase of interest relatively quickly, especially in comparison to the context speech, which increases the likelihood of hearing *for*, and could explain why he was adamant that he said *for a*.

Syntax is also a potential source of information for disambiguating what Armstrong said. Mattys and colleagues [20] demonstrated that syntactic information can help to disambiguate acoustic information regarding where word boundaries are present in speech, and therefore determine whether *for a* or *for* was spoken. It turns out that Armstrong’s quote is syntactically ambiguous in the vicinity of the region in question, further complicating the quote’s interpretation. The syntactic context *for (a) man* does not disambiguate whether the function word is present or not, since “man” can refer to mankind or to a single, male individual. Thus, context speech rate may be an even more important cue for listeners.

This prior research suggests that the case of ambiguity in the famous quote by Armstrong represents the *perfect storm* of conditions for misperception; though a function word may have actually been spoken, it was not perceived due to a number of factors, including possible reduction and coarticulation of *for a*, in addition to an ambiguous syntactic context. Furthermore, the region of interest, *for (a)*, in the original quote is quite short (127 ms) and may be more acoustically consistent with typical productions in which a speaker produces only the word *for*.

In the present study, we explore two time-based causes of the ambiguity in Armstrong’s quote. We tested the hypothesis that the temporal properties of naturally produced, reduced tokens of *for* (spoken as “fer”, [fɚ]) followed by *a* significantly overlap with those of *for* (spoken as “fer”, [fɚ]) not followed by the word *a*, leading to significant ambiguity about the presence or absence of the word *a*. In Experiment 1, we examine whether productions of *for a* and *for* are similar in duration in casual speech that *for a* can easily be mistaken for *for*. In Experiment 2, we ask whether these naturally produced tokens are susceptible to the “disappearing word effects” originally demonstrated in a less spontaneous form of speech [14]. Similar to the case of Armstrong’s original phrase, in which a slow surrounding context speech rate results in a short duration of the phrase *for (a)* relative to its context, we hypothesized that when tokens of speech including a reduced string of *for a* had context speech slowed down relative to the *for a*, the function word *a* would be less likely to be reported.

## Experiment 1: Production Study

### Methods

Tokens were extracted from the Buckeye Corpus for Conversational Speech [21], which contains spontaneous speech from 40 talkers (20 female, 20 male) from the Columbus, Ohio area, near Armstrong’s hometown of Wapakoneta, Ohio. We used the orthographic and phonetic transcriptions provided with the Buckeye Corpus to discriminate between cases of *for* versus *for a*, rather than determining whether there were spectral traces of *a* after *for*. The procedures for data collection of this corpus were approved by the Ohio State University Institutional Review Board and all participants in the corpus provided informed consent in writing.

Each instance of *for* followed by *a* (i.e., a “*for a* token”) was extracted from the corpus (a total of 191 tokens). Each token was matched with a production of *for* followed by a noun (a “*for* token”), spoken by the same speaker; resulting in an additional 191 tokens. In all cases, the selected *for* and *for a* tokens were spoken in a reduced style, e.g., cases where *for* was transcribed in the Buckeye corpus as “fer” [fɚ] [22]. While it is possible that transcription accuracy may be subject to perceptual effects, there are relatively few cases in which *for* and *for a* are locally ambiguous. Nearly all of those cases are resolved in the broader context, making transcription errors due to ambiguity relatively unlikely. We matched the word following the target region exactly whenever possible, though this matching was only possible in about 17% of cases. We also tried to roughly match for prosodic characteristics (i.e., word stress and position relative to the end of a prosodic phrase); such matches were possible in over 90% of cases. It should be noted that *a* is a clitic function word, meaning it is phonologically dependent on another word. We do not differentiate between proclitic and enclitic forms in this analysis; rather, we take a more agnostic stance with regard to this reduction phenomenon, analyzing all instances of cliticization of *a* that met our other criteria, above.

The target region for durational measurement was defined as the words *for a* or *for*. The target region was defined as the start of /f/ to the beginning of the following noun or adjective, either after the word *for* or the phrase *for a*. Normalized durations were calculated by dividing the duration of the target region by the average duration of the two preceding syllables, to provide a rough normalization for the local speech rate. The duration of [fɚ] in “for (a) [man]” from the original recording of Armstrong’s lunar transmission was also measured. We used permutation tests to compare the distribution of the *for* tokens to the distribution of the *for a* tokens. The permutation test (or randomization test) uses resampling techniques to determine all possible distributions of the test statistic rather than assuming a distribution, as in the case of most parametric statistics [23]. Permutation tests do not require normality assumptions or that distributions be of equal sample size. They provide a means of assessing how similar two distributions are. As with all resampling methods, there is the strong assumption that the samples themselves are representative of the populations. There is no reason to think ours are unrepresentative, and indeed, in spoken corpus work, there are few other sources with as much variety of speech across talkers and age. Each permutation test used 1000 iterations to resample the data.

### Results and discussion

**Fig 1:**
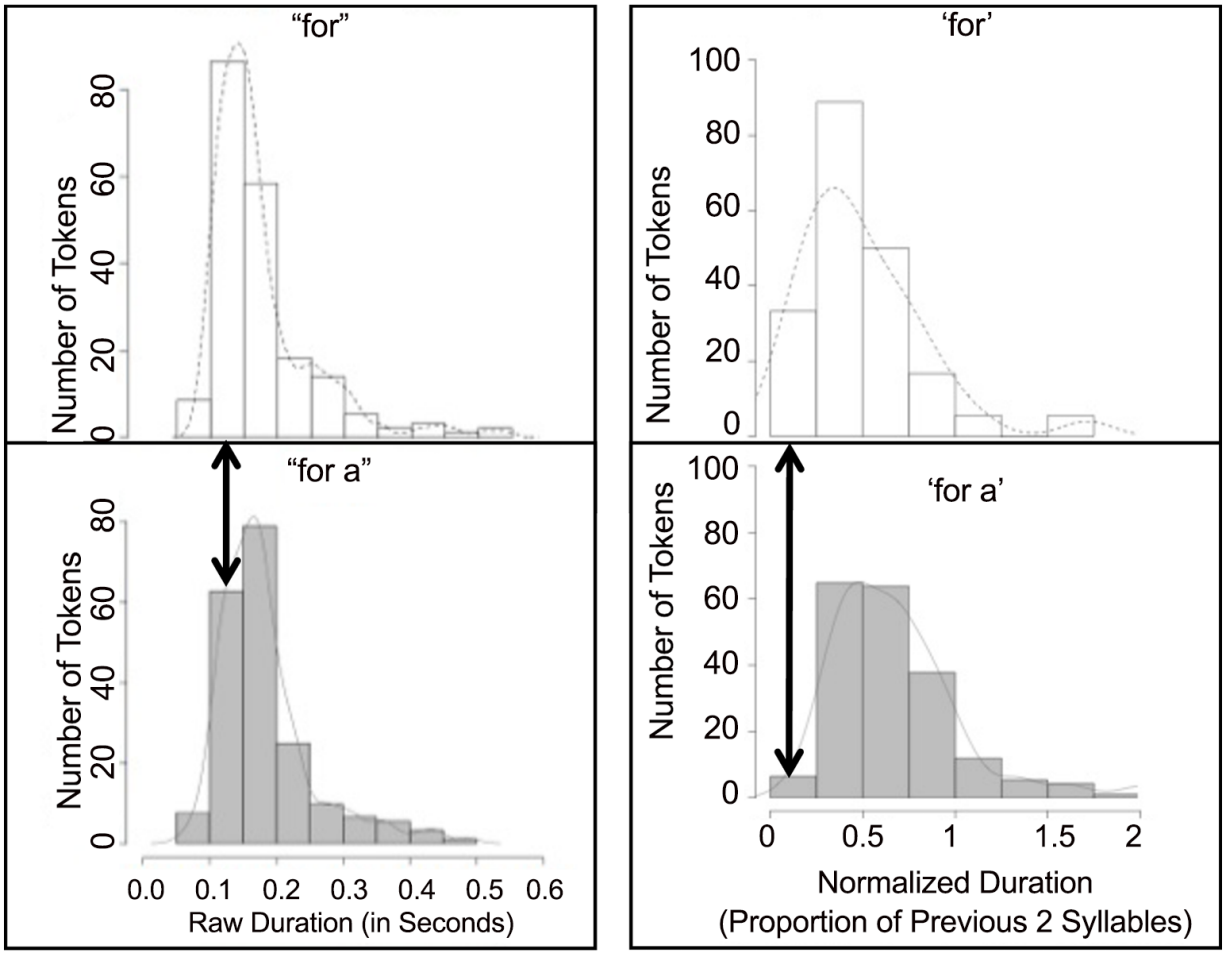
**(a—left)** Number of tokens of *for* and *for a* as a function of duration. **(b—right)** Number of tokens of *for* and *for a* as a function of normalized duration. Superimposed lines represent a smoothed function. The arrow indicates the location of Armstrong’s original production within the distribution of tokens examined here. Fig 1. a. is reprinted from Dilley, L., Baese-Berk, M. M., Schmidt, S., Nagel, J., Morrill, T., & Pitt, M. One small step for (a) man: Function word reduction and acoustic ambiguity. Proc Meet Acoust. 2013; 19: 060297. under a CC BY, license, with permission from the Acoustical Society of America, original copyright 2013.

As shown in Fig 1a, distributions of the durations of the target regions for *for a* tokens and *for* tokens are very similar. Measures of normalized duration (Fig 1b), using the speech rate of the surrounding context, also yield similar patterns. There was no statistically significant difference between the duration of the target region for *for* tokens and *for a* tokens (*p* = 0.56). The average duration of *for* tokens was 168 milliseconds (s.d. = 53) and the average duration of *for a* tokens was 225 milliseconds (s.d. = 66).

Examining the original recording of the lunar transmission revealed that the analogous region in Armstrong’s original quote, *for (a)*, was 127 milliseconds. Based on the distribution of duration information in the representative samples of *for* and *for a* tokens from talkers with a similar dialect in Ohio, the durational information of [fɚ] in Armstrong’s original recording is highly compatible with either *for a* or *for* interpretations of the ambiguous stretch of speech (Fig 1a). However, the duration is slightly more compatible with the *for* distribution. We discuss the implications of this below.

**Fig 2:**
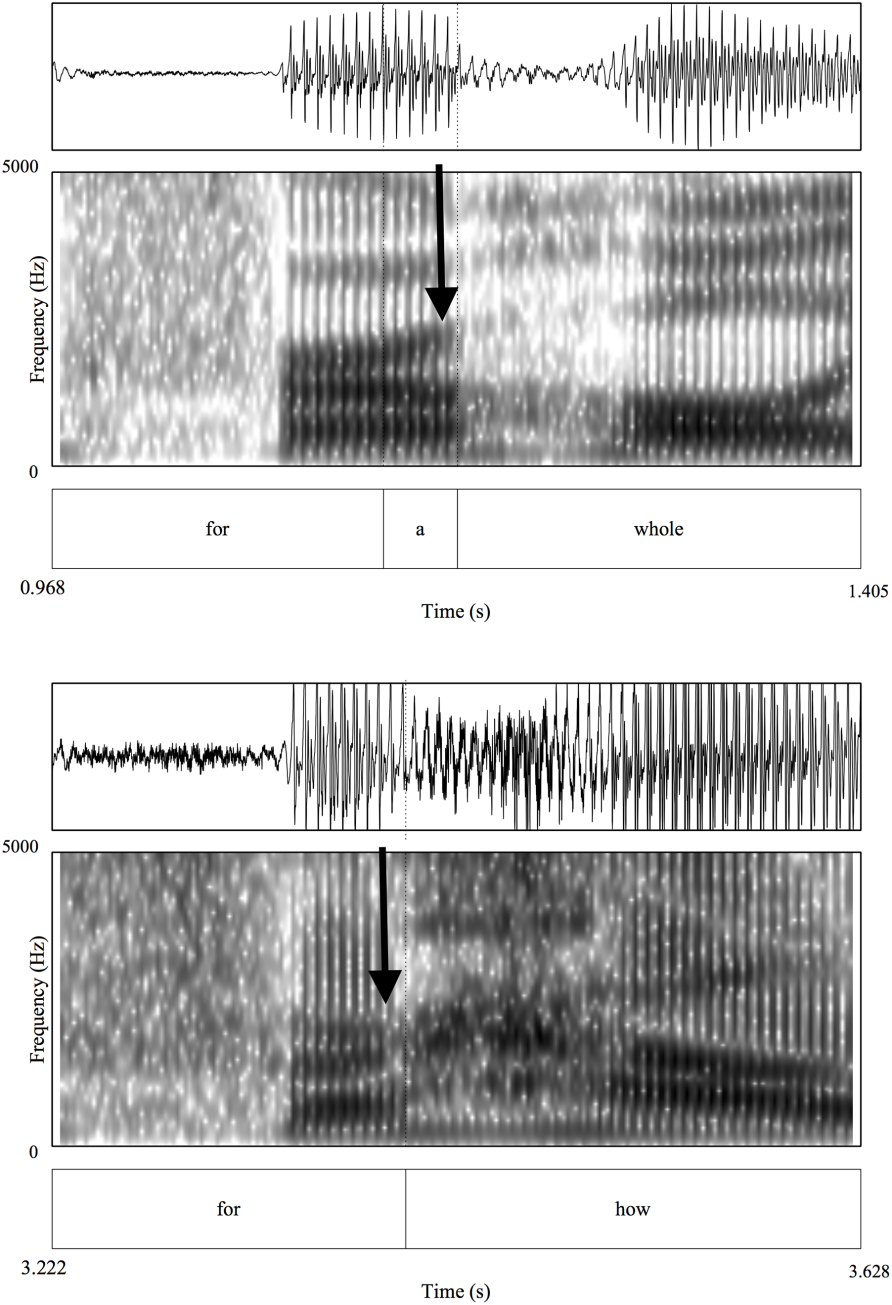
**(a—top)** Production of *for a whole [year]* spoken by a single speaker in the Buckeye corpus **(b—bottom)** Production of *for how [long]* spoken by the same speaker in the Buckeye Corpus. Arrows indicate specific areas of interest for formant movement in F2 and F3.

It is important to note that spectrally these two sets of tokens are also quite similar. In all tokens, *for* was reduced to [fɚ], resulting in substantial spectral overlap, even in cases when followed by the function word *a*. In order to further investigate spectral similarity, we extracted formant values for the second and third formants of the target regions. Specifically, we examined the separation between F2 and F3 at 15%, 50% and 85% of the duration of vocalic region. The rhotic segments /ɹ/ and /ɚ/ are associated with a particularly low F3 value [24], which distinguishes them from non-rhotic segments (e.g., */*ə/). Thus, if *a* is present spectrally, in spite of a very similar duration, then there would have been an articulatory movement away from the rhotic sound in *for*, resulting in an increased F3 and effectively a larger separation between F2 and F3 late in the vocalic portion of *for a* tokens compared with *for* tokens. However, there should be no difference in the magnitude of the separation between F2 and F3 early in the word for the two token types. This is, in fact, what we found. As in the case of duration, we used permutation tests to examine the statistical significance of the difference in spectral qualities of the tokens. There is no significant difference in the size of the separation between F2 and F3 at the 15% point in the target regions (15% *for* mean = 493 Hz; 25% *for a* mean = 513 Hz, *p* = .78). However, there is a small but significant difference in the size of the separation between F2 and F3 for *for* and *for a* tokens at the 50% and 85% points in the tokens (50% *for* mean = 462 Hz; 50% *for a* mean = 530 Hz, *p* < .02; 75% *for* mean = 490 Hz; 75% *for a* mean = 835 Hz, *p* < .001). This suggests that while the two types of tokens are quite similar in terms of duration, there are some spectral differences that may allow listeners to disambiguate them. Fig 2 presents data from the same speaker producing *for a whole [year]* and *for how [long]*. There is a separation between F2 and F3 and an F3 rise that occurs at the end of the vocalic region in *for a whole* that is not apparent in *for how*.

While the duration of Armstrong’s production results in a potentially ambiguous percept, listeners typically have no trouble determining which of multiple options they heard given an ambiguous target. The distributions of *for* and *for a* suggest that if listeners were required to determine which of multiple options they heard, and they were to rely predominantly or wholly on duration in judging the lexical content of this portion of the phrase, a duration of 127 milliseconds would be more likely to be interpreted as *for* (with no following *a*) than as *for a*. Armstrong’s token is in the 3^rd^ percentile of the *for a* distribution but the 6^th^ percentile of the *for* distribution. Further, when examining the duration of the token as a function of the previous two syllables, which were spoken relatively slowly (1059 milliseconds), the resulting proportion is, statistically speaking, slightly more likely to be interpreted as *for* rather than as *for a*. The separation between F2 and F3 in Armstrong’s original token was 275 Hz, which is quite small compared to the average of either distribution, suggesting that even if listeners combined spectral information with the duration information in the token the possible percept was still ambiguous.

The results of Experiment 1 suggest that naturally produced tokens of *for* and *for a* contain substantial overlap and create an acoustically ambiguous environment for lexical access. While this percept is slightly more consistent with an interpretation of *for*, either option is consistent with the distributions seen in natural productions. In Experiment 2, we ask whether another aspect of Armstrong’s original productions, i.e., the relatively slow context speech rate, may influence the perception of *for* vs. *for a*.

## Experiment 2: Perception Study

### Methods

35 students from Michigan State University participated in the study. All were native American English speakers with no reported learning or speech disabilities. Though we did not collect a detailed questionnaire about where the students were from or their exposure to other dialects, it is likely that participants had wide experience with the dialect spoken by talkers in the Buckeye Corpus, given that Michigan and Ohio are bordering states, part of the same Midwestern region, and many students from Ohio attend Michigan State University. Data collection procedures for this experiment were approved by the Institutional Review Board at Michigan State University. All participants provided written consent to participate in the experiment.

Stimuli for this study were tokens from Experiment 1 which contained syntactically ambiguous productions similar to Armstrong’s original production. We chose to use the *for a* tokens rather than the *for* tokens because in the original study by Dilley and Pitt [14], the context speaking rate effect was found to be substantially larger for the cases in which a function word was produced in the original stimulus than in cases where the function word was not produced. We determined syntactic ambiguity by judging the lexical content of each individual token and deciding where it would be grammatically acceptable for *a* to be omitted. Eleven ambiguous *for a* tokens were chosen (e.g., “*Y’know I was there just* ***for a*** *half a day and”* in which *a* is optional for the sentence to be well-formed). Two target tokens contained an additional instance of *a*. Participants also heard 33 filler tokens that did not include *for* or acoustically ambiguous regions.

Following Dilley and Pitt [14], tokens were presented in slowed or unaltered context speech rate conditions. All target stimuli were divided into a target and context region. The target region consisted of *for a*, the syllable before and the phoneme immediately following. The context region included the rest of the token, before and after the target region. For example, in the sentence, “*Y’know I was there* *just for a h**alf a day and*”, the target region consisted of the underlined portion “*just for a h-*” and the rest of the sentence before and after the target region was considered the context region. Word and phoneme level alignment from the Buckeye Corpus was used to determine the target and context regions (see Pitt et al., 2007 for more details on alignment within the corpus). For the slowed rate condition, the context region was time expanded by a factor of 1.75 using the PSOLA algorithm in Praat [25]. Tokens in the unaltered rate condition were multiplied by 1.0 using the PSOLA algorithm, maintaining their original speech rate. Filler items were presented at either the unaltered or slowed rate; the duration of the entire utterance was manipulated.

Each participant listened to one of two counterbalanced lists that consisted of the eleven *for a* tokens and the fillers in a randomized order. In each list, the tokens were presented in either the unaltered or slowed condition. After listening to each token, the listeners were asked to transcribe the sentence using the keyboard.

We analyzed the transcriptions to determine whether listeners reported hearing *for a* in the critical trials. Responses were analyzed using mixed-effects logistic regression. The model predicted the likelihood of the listener reporting *a*; context speech rate and the separation between F2 and F3 and their interaction were included as fixed factors. The model included the maximal random effect structure that would allow the models to converge and included random intercepts for subjects and items [26].

### Results and Discussion

The transcriptions of the *for a* tokens yielded higher reports of *for a* in the unaltered condition than in the slowed condition (*β* = -1.22, s.e. (*β) =* .496, *z* = -2.458, *p* < .015). Though all targets included *a*, listeners were less likely to report hearing *a* when the context speaking rate was slower than in its unaltered state, replicating the “lexical rate effect” from Dilley and Pitt [13]. Listeners reported not hearing *a* on 20.8% of trials in the unaltered condition and on 40.2% of trials in the slowed condition. The interaction between speaking rate and the difference between F2 and F3 was not significant (*β* = -0.0005, s.e. (*β) =* .001, *z* = -0.56, *p* = .576). Interestingly, however, the difference between F2 and F3 was a significant predictor of hearing *a* (*β* = 0.018, s.e. (*β) =* .0008, *z* = 2.399, *p* < .017). This suggests that both the difference between F2 and F3 and the context speaking rate of the sentence influence the perception of the target, consistent with previous experimental work by Heffner et al. [14]. That is, both fine-grained phonetic detail and more global phonetic information influence perception.

These results support the hypothesis that when target speech containing *for a* is relatively fast compared with a (slower) context—similar to conditions apparently present in Neil Armstrong’s speech—listeners tend to perceive one word—*for—*rather than two words—*for a*. Context speech rate is particularly important when listeners cannot rely on linguistic knowledge [16,27]. Durational information is so important in ambiguous spectral contexts that changes in relative timing information can cause a word in a speech signal to completely disappear perceptually. It is possible that listeners who heard Neil Armstrong’s quote would have reported not having heard *a*, even if the spectral information was non-ambiguous, because the context speech rate was relatively slow. If the context had been faster relative to the target portion, it is possible listeners would have reported hearing *for a*, rather than *for*.

## General Discussion

The motivation of the current study was to investigate cases of ambiguity in temporal and spectral characteristics of spontaneous, naturally-occurring speech. This work addresses an important topic in current psycholinguistic research—production of reduced tokens and how listeners recover perceptual information from reduced speech [6,10–13]. Additionally, this study contextualizes Neil Armstrong’s famous quote and his reports that the words he spoke were different than those perceived. Specifically, we hoped to examine possible causes for this mismatch and use the case of this famous quote as a case study for the importance of speech rate in models of spoken word recognition. We hypothesized that productions of *for* and *for a* may not be consistently differentiated by duration or spectral cues. Further, we hypothesized that listeners may use context speech rate to disambiguate between the two options potentially ambiguous, an idea consistent previous findings on context speech rate [14]. In Experiment 1, we demonstrate that the duration profile of *for* and *for a* is highly similar with a great deal of overlap between the distributions of naturally produced tokens of each phrase. In Experiment 2, we demonstrate that the relative rate of the target and context speech affected the proportion of *for a* versus *for* reports. In the unaltered condition, listeners reported hearing *for a* more frequently than in the slowed condition.

Studies have previously examined how timing information is used in speech perception, resulting in the generalized rate-normalization hypothesis [14]; this relates the duration of a stretch of speech relative to its surrounding context speech with the number of perceived lexical units in that stretch. Dilley and Pitt demonstrated the extent to which context speech rate is used by listeners to interpret a spectrally and grammatically ambiguous speech signal. The current studies provide support for this hypothesis. Because productions of function words frequently result in spectral and temporal overlap, listeners use speech rate to differentiate between these ambiguous regions, presumably by relating the target region to the duration of the context speech in order to determine the amount of lexical material in the ambiguous region.

It is important to note that the raw duration of the target region was identical across conditions. Therefore, the percept reported by participants was not due to the raw duration of the target region, but rather the relationship between the duration of the target material and the rate of speech preceding the target, suggesting that this result can be attributed to solely *relative* temporal information. Therefore, it is possible that if the original phrase spoken by Neil Armstrong contained a relatively short target region, *for (a)*, and a relatively slow context speech rate, this factor could contribute to the percept of *for*. While it is possible that semantic properties of this production were more consistent with an interpretation of *for a*, nevertheless both interpretations were possible, and the acoustic properties—both local and distal—may have overridden the default semantic interpretation. Overall, the results of the two studies here demonstrate that speech can be highly ambiguous such that the relationship of relative speech rate between a target phrase and the surrounding context speech influences perception; relative duration can cause a produced word to perceptually disappear.

These results suggest that Neil Armstrong’s statement could have been a “perfect storm” of conditions making the listener more likely to perceive *for* rather than his intended *for a*. was His production of *for [a]* was durationally ambiguous, falling within the normally-used durational and spectral cues of both *for* and *for a* productions. In addition, his statement was syntactically ambiguous. Further, it is impossible to know what Armstrong himself heard during his production, and how that feedback, or lack thereof, may have influenced his production. However, it is possible that listeners who heard his quote and reported hearing *for* rather than *for a* did so because of inconsistencies in his speaking rate, and a different relationship between the context and the production of *for [a]* would have rendered greater agreement between his claim and those who heard his speech. Perhaps, if only he had produced the target region more slowly, Armstrong’s quote would have been perceived as he claimed to have intended it. The present study is a case study of a fundamental, pressing psycholinguistic problem regarding the challenge faced by listeners in recovering a speaker’s intended message. This challenge is particularly difficult when the signal for that message contains ambiguous, highly reduced phonetic information and syntactic and semantic knowledge alone cannot disambiguate the signal. The current results emphasize the importance of timing information in this recovery process and in perception and spoken word recognition generally speaking.
